# FIV vaccine with receptor epitopes results in neutralizing antibodies but does not confer resistance to challenge

**DOI:** 10.1038/s41541-018-0051-y

**Published:** 2018-04-30

**Authors:** Craig Miller, Mauren Emanuelli, Elizabeth Fink, Esther Musselman, Ryan Mackie, Ryan Troyer, John Elder, Sue VandeWoude

**Affiliations:** 1 Department of Microbiology, Immunology, and Pathology, Colorado State University, Fort Collins, CO USA; 2 Department of Immunology and Microbiology, The Scripps Research Institute, La Jolla, CA USA

## Abstract

Feline immunodeficiency virus (FIV) is the feline analogue to human immunodeficiency virus (HIV) and utilizes parallel modes of receptor-mediated entry. The FIV surface glycoprotein (SU) is an important target for induction of neutralizing antibodies, and autoantibodies to the FIV binding receptor (CD134) block infection ex vivo; thus highlighting the potential for immunotherapies which utilize anti-receptor antibodies to block viral infection. To determine whether vaccination with CD134-SU complexes could induce protection against FIV infection, cats (*n* = 5 per group) were immunized with soluble CD134, recombinant FIV-SU protein, and/or CD134+SU complexes. Two trials were performed with different antigen combinations and vaccination schedules. In vivo generation of anti-CD134 and anti-SU IgG antibodies was measured, and in vitro neutralization assays were conducted. Immunization induced production of anti-CD134 and anti-SU antibodies that significantly inhibited FIV infection in vitro. However, no vaccine combination protected cats from FIV infection, and neat serum from vaccinated cats enhanced FIV growth in vitro. CD134+SU vaccinated cats exhibited increased CD4:CD8 ratio immediately prior to challenge, and antibodies were much more efficiently generated against vaccine by-products versus target antigens. Results suggest vaccination against viral and cryptic receptor epitopes yields neutralizing antibodies that synergistically inhibit FIV infection in vitro. Factors contributing to vaccine failure may include: (1) Heat-labile serum factors that enhance viral replication, (2) changes in circulating target cell populations induced by vaccination, and (3) weak immunogenicity of neutralizing epitopes compared to off-target vaccine components. Results reinforce the need to monitor vaccine preparation components and avoid non-specific immune stimulation during vaccination.

## Feline immunodeficiency virus: In vivo protection remains elusive

A vaccine candidate for feline immunodeficiency virus elicits strong immunological reaction in vitro, but no protection to live cats. The feline analog to human immunodeficiency virus, FIV shares a similar infection paradigm and has only one partially effective vaccine. A US team, led by Colorado State University’s Susan VandeWoude, immunized cats using a complex of an FIV surface protein and a feline cell-surface protein known to facilitate FIV’s entry into immune cells. Tissue culture assays yielded promising results; however, this did not translate to live-animal protection. The researchers highlighted multiple factors that could explain the lack of success, including circulatory pro-infection factors, and immune responses generated against vaccine by-products rather than intended targets. While the vaccine candidate failed, the research provides invaluable guidance for future efforts into FIV vaccination with implications for HIV vaccine trials.

## Introduction

**Fig. 1:**
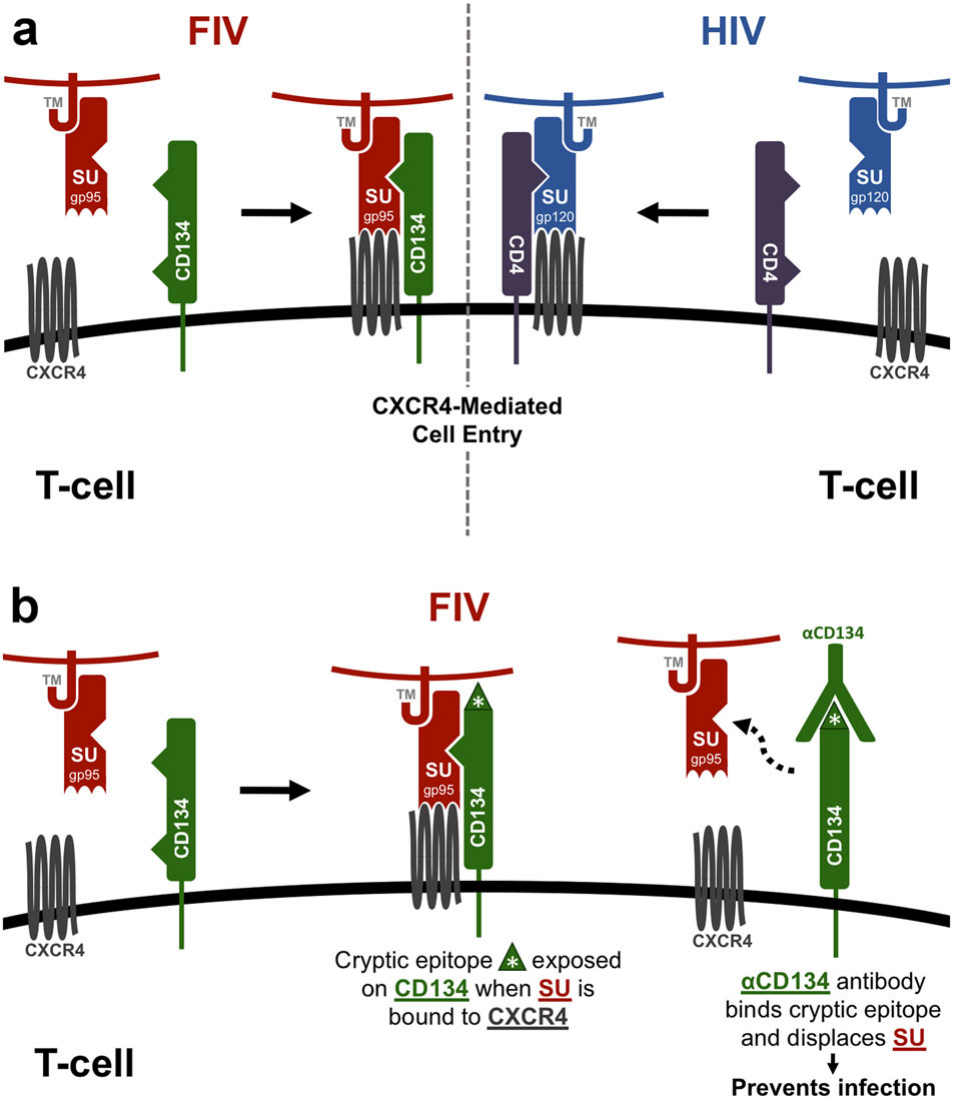
**a** FIV and HIV utilize analogous modes of receptor-mediated cell entry. HIV binds to CD4+ target cells through a high-affinity interaction with the CD4 receptor, inducing a conformational change in the envelope glycoprotein gp120 (SU) that exposes the CXCR4 co-receptor binding site and subsequent fusion with the cell membrane. FIV utilizes a primary receptor CD134, and similar to HIV, binding of FIV Env to the CD134 receptor alters the conformation of envelope glycoprotein gp95 surface (SU) component to facilitate CXCR4 co-receptor binding, and viral entry. **b** Anti-CD134 antibodies prevent FIV infection in the presence of viral glycoproteins. Binding of FIV SU to CD134 induces a conformational change in the receptor and exposes a cryptic epitope that results in anti-CD134 generation. Anti-CD134 antibody binding to CD134 induces a second conformational change in the CD134 receptor that displaces SU from the cell surface, resulting in inhibition of infection.^31^

Feline immunodeficiency virus (FIV) is a naturally occurring lentivirus that is genetically similar to human immunodeficiency virus (HIV) and shares many immunopathogenic features of HIV infection.^1–6^ Like HIV, FIV primarily infects and replicates within CD4+ T cells, and is characterized by progressive depletion of CD4+ T lymphocytes and an AIDS-like syndrome during natural infection of domestic cats.^7–9^ Both lentiviruses require an initial interaction with a primary binding receptor for infection, and utilize analogous modes of receptor-mediated entry via the chemokine co-receptor, CXCR4.^10–12^ HIV binds to CD4+ target cells through a high-affinity interaction with the CD4 receptor that induces a conformational change in the envelope glycoprotein gp120 to expose binding sites necessary for chemokine co-receptor binding (CXCR4 or CCR5) and subsequent fusion with the cell membrane (Fig. 1a).^10,11^ FIV utilizes CD134 as primary binding receptor, and studies have demonstrated that binding of the CD134 receptor alters the conformation of FIV envelope protein gp95 (SU) in a similar fashion to that, which occurs in the CD4/HIV gp120 interaction, to promote high-affinity binding with the entry receptor CXCR4 (Fig. 1a).^13,14^

Since the discovery of FIV, considerable effort has been directed at the development of safe vaccine strategies that can produce protective immunity in cats.^15,16^ A commercially available, whole-inactivated virus vaccine containing two FIV subtypes (Fel-O-Vax FIV®) is currently licensed for use in the United States, and various reports have described virus neutralization and cellular immunity in a significant proportion of study animal.^17–20^ However, the efficacy of this vaccine is still under debate, as recent studies and field evaluations have reported that the vaccine does not confer immunity against certain FIV strains (i.e.: FIV_GL8_), and that the neutralizing antibody response and protective rate may be low in certain cat populations (i.e., protection is not conferred to certain virulent recombinant strains of FIV).^21–24^ Other attempts at FIV vaccine development have either failed to induce protective immunity against FIV infection, or have resulted in increased susceptibility to infection via antibody-dependent enhancement or general immune activation.^25–30^ Thus, the development of immunotherapies aimed at preventing viral entry may have great potential to increase the level of protection against naturally occurring FIV infection.

Previous studies by Grant et al.^31^ reported that antibodies to the CD134 receptor (anti-CD134) and the viral surface glycoprotein (anti-SU) are expressed in a high proportion of chronically FIV-infected cats, and that increased levels of anti-CD134 are correlated with lower viral loads and improved health status in these animals.^31^ Furthermore, anti-CD134 antibodies purified from serum of infected cats have been shown to exhibit significant neutralizing activity and are able to block FIV infection ex vivo.^31^ This activity is related to binding of FIV SU to CD134 on the target cell, inducing a conformational change that exposes a cryptic epitope of CD134 that is recognized and blocked by feline anti-CD134.^31^ Binding of anti-CD134 autoantibodies to CD134 causes a second conformational change in the CD134 receptor and results in displacement of SU from the cell surface (Fig. 1b), demonstrating an active contribution of anti-receptor antibody responses to controlling viral infection.^31^ Parallel mechanisms of cell entry are also observed in HIV infection, whereby neutralizing epitopes are exposed following interaction of SU with the CD4 binding receptor.^32–36^ Thus, the analogous modes of receptor-mediated viral entry that exist between FIV and HIV likely reflect common immunological pressures in the two hosts, and may indicate a convergent pathway for the development of strategies to compromise the virus’ ability to escape immune surveillance.^2^

Such propensity for anti-CD134 and anti-SU antibodies to block FIV infection ex vivo, coupled with the increased survival of cats expressing high levels of anti-CD134 antibodies, highlights the potential for immunotherapies, which utilize anti-receptor antibodies to protect from viral infection. In this study, we assessed the potential for immunization with soluble CD134 and FIV-SU complexes (CD134+SU) to induce a neutralizing antibody response and protection against FIV infection in domestic cats. In Protocol I of this study, cats were immunized with FIV-PPR-SU-huFc immunoadhesins, either alone (**SU**-huFc group) or together as a complex with soluble CD134-huFc (**CD134+SU**-huFc) (SI Fig. 1A). Protocol II of this study employed soluble CD134 and FIV-PPR-SU derived by expression and purification from human 293ST cells, and evaluated whether immunization with soluble CD134-293S and FIV-PPR-SU-293S immunoadhesins, either alone (**CD134**-293S) or together as a complex (**CD134+SU-**293S), would augment the efficacy of a vaccine. Our results demonstrate that immunization with soluble CD134 and recombinant SU protein induces production of anti-CD134 and anti-SU antibodies, and that these antibodies significantly inhibit FIV infection in vitro. However, vaccinated cats became infected following FIV challenge, and vaccination altered circulating cell populations with potential consequences for subsequent infection. Furthermore, antibodies were generated against irrelevant antigens in the vaccine preparation at levels that significantly exceeded antibodies produced in response to target antigens. This study highlights potential targets and requirements for vaccine optimization for anti-receptor and anti-immunodeficiency virus development.

## Results

### Anti-CD134 and anti-FIV-SU antibodies generated in vivo prior to viral challenge

#### Protocol I

**Fig. 2:**
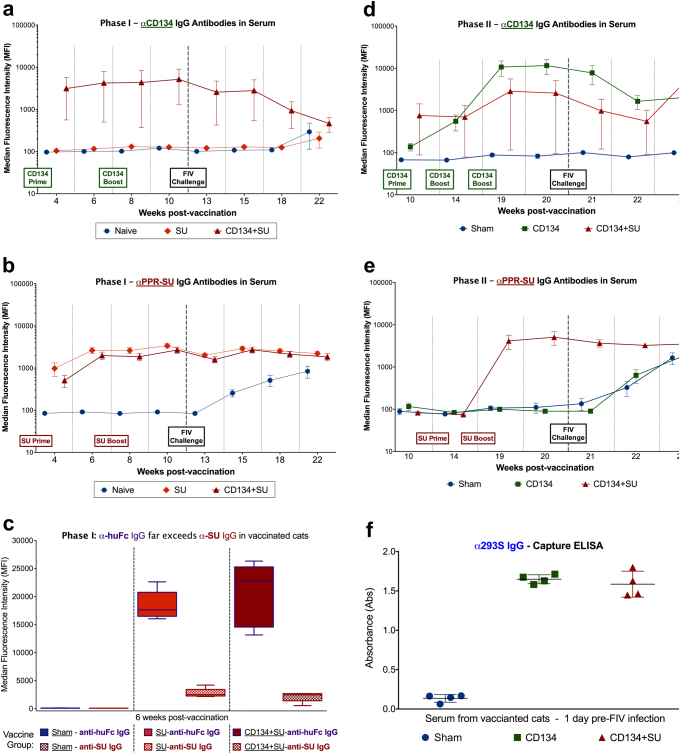
Anti-CD134, anti-SU, and irrelevant antibodies (α-huFc, α-293S) are elicited during vaccination. **a** Microsphere immunoassay (MIA) detected anti-CD134 IgG antibodies in CD134+SU vaccinated cats beginning at week 4 post vaccination and peaking at 10 weeks post vaccination (3 weeks after final CD134 boost). **b** Anti-SU IgG was detected by MIA in SU vaccinated and CD134+SU vaccinated cats beginning at week 6 post vaccination and these levels differed significantly over time compared to Sham vaccinated controls (*p* < 0.0001). **c** MIA measured IgG antibodies against huFc tag used to purify soluble CD134 and PPR-SU rProteins in Protocol I vaccine preparation. Anti-huFc IgG levels (indicated by MFI) significantly exceeded levels of anti-SU and anti-CD134+SU (*p* < 0.0001). Relative concentration of antibody response to huFc was estimated to be 10-fold higher than anti-SU specific IgG response (as described in text). **d** MIA detected significant quantities of anti-CD134 IgG antibodies in serum from CD134 and CD134+SU vaccinated cats beginning at week 14 post vaccination (6 weeks pre-FIV infection) which increased significantly over time in CD134 vaccinated cats compared to sham vaccinated background levels (*p* = 0.003). **e** Anti-SU IgG antibodies were detected in CD134+SU vaccinated cats by MIA at 1 week prior to FIV challenge (2 weeks after final SU boost), and antibody levels increased significantly over time compared to controls (*p* = 0.001). **f** Capture ELISA detected high levels of anti-293S antibodies in serum of both CD134 vaccinated (*p* < 0.0001) and CD134+SU vaccinated (*p* < 0.0001) cats immunized with the 293S-purified CD134 and/or FIV-SU vaccine construct compared to sham vaccinated cats

Serum samples from weeks 4, 6, 8, 10, 13, 15, 18, and 22 were tested by microsphere immunoassay (MIA) to detect anti-SU and/or anti-CD134 IgG antibodies in cats vaccinated with FIV-PPR-SU-huFc or a combination of CD134+SU-huFc; results of which are presented in Fig. 2. Increased levels of anti-CD134 antibodies were detected as early as week 4 post vaccination in CD134+SU-huFc vaccinated cats and were significantly elevated at week 19 (1 week pre-FIV infection) compared to background levels (treatment *p* = 0.014), but decreased slightly after infection (Fig. 2a). As expected, anti-CD134 IgG antibodies were not detected in serum from SU-huFc vaccinated or sham vaccinated study animals prior to FIV-infection (Fig. 2a).

Increased levels of anti-SU IgG were detected in SU-huFc vaccinated and CD134+SU-huFc vaccinated cats beginning at week 4 post vaccination (Fig. 2b), and these levels differed significantly over time when compared to background levels (interaction *p* < 0.0001). Anti-SU IgG antibody levels remained elevated in all SU-huFc and CD134+SU-huFc vaccinated animals after FIV infection. Anti-SU IgG antibodies were not detected in sham vaccinated cats prior to FIV infection, but increased slightly following intravenous viral inoculation as expected (Fig. 2b). When serum samples were evaluated for antibodies against the huFc tag used to purify the soluble CD134 and PPR-SU rProteins in the vaccine preparation, significantly increased levels of anti-huFc IgG were detected in SU-huFc and CD134+SU-huFc vaccinated cats (*p* < 0.0001) which often exceeded anti-SU IgG production by more than 10-fold (Fig. 2c).

#### Protocol II

Serum samples from weeks 10, 14, and 19–23 were tested by MIA to detect anti-CD134 and anti-SU IgG antibodies in cats vaccinated with soluble CD134 and PPR-SU rProteins purified from 293S cells. Anti-CD134 IgG antibodies were detected in serum from CD134 vaccinated cats beginning at week 14 post vaccination (6 weeks pre-FIV infection) and increased significantly over time compared to sham vaccinated background levels (interaction *p* = 0.003) (Fig. 2d). Similarly, anti-CD134 IgG was detected in CD134+SU vaccinated animals beginning at week 10 post vaccination (10 weeks prior to FIV infection), but the anti-CD134 IgG response was not as robust in this vaccine group and did not differ significantly from background levels (interaction *p* = 0.135) (Fig. 2d). Anti-SU IgG antibodies were detected in CD134+SU vaccinated cats beginning at week 19 (1 week prior to FIV infection), and antibody levels increased significantly over time compared to the background levels (interaction *p* = 0.001) (Fig. 2e). As expected, anti-SU antibodies were not detected in sham vaccinated or in CD134 vaccinated cats prior to FIV infection, but increased slightly over time after intravenous viral inoculation. Similar to Protocol I, high levels of anti-293S antibodies were detected by ELISA in serum of cats vaccinated with 293S-purified soluble CD134 and/or PPR-SU rProteins (Fig. 2f). Specifically, serum from CD134 and CD134+SU vaccinated cats exhibited significantly elevated absorbance (Abs) values compared to serum from sham vaccinated cats (interaction, *p* < 0.0001), indicating that significant in vivo antibody production occurred in response to the 293S cell products used in vaccine production.

### FIV challenge

#### Protocol I

**Fig. 3:**
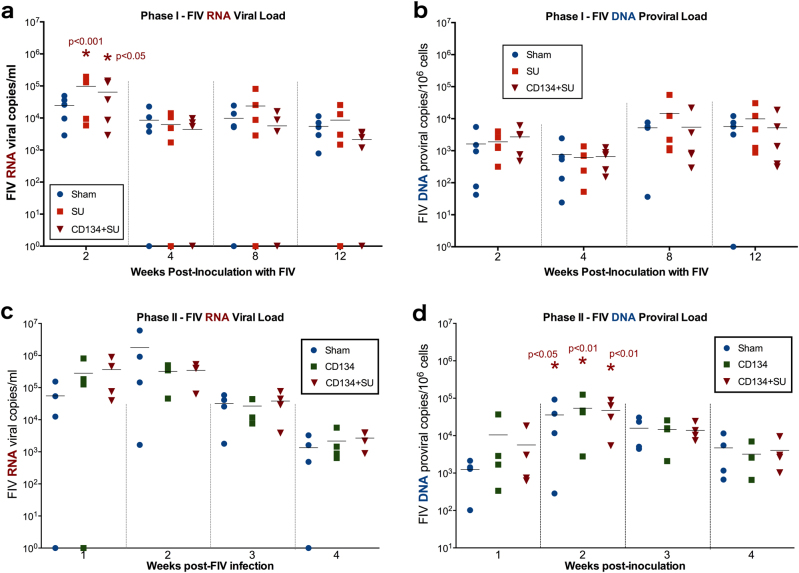
CD134 and/or SU vaccinated cats were not protected against FIV challenge. **a** All Protocol I study animals demonstrated positive viremia by week 2 post FIV infection, and FIV RNA levels were significantly elevated at week 2 in SU vaccinated (*p* < 0.001) and CD134+SU vaccinated (*p* < 0.05) cats; suggesting a transient enhancement of FIV infection. **b** Proviral DNA was detected in PBMCs of all Protocol I study animals by week 2 post FIV infection, but did not differ between vaccine groups over time. **c** All Protocol II study animals demonstrated FIV RNA in plasma by week 2 post infection. FIV RNA levels decreased significantly over time in all groups (time *p* < 0.001); however, no significant differences were observed between vaccine groups over time during this phase of the study. **d** Proviral DNA was detected in PBMCs of all Protocol II study animals at week 1 post FIV infection, and were significantly elevated above baseline (pre-FIV challenge, week 20) in all study groups by week 2 post FIV infection (RM-ANOVA). Proviral DNA levels did not differ over time or between vaccine groups during Protocol II

At week 12 post vaccination, study animals were intravenously inoculated with FIV_PPR_ as described above. Plasma samples from all study animals in all vaccine groups had detectable FIV plasma RNA by week 2 post FIV infection (Fig. 3a). Moreover, FIV RNA levels were significantly elevated at week 2 in SU-huFc vaccinated (*p* < 0.001) and CD134+SU-huFc vaccinated (*p* < 0.05) study animals when compared to sham vaccinated animals (Fig. 3a), suggesting a transient enhancement of FIV infection in these vaccine groups. PBMC FIV proviral DNA was detected in all study animals by week 2 post FIV infection, and proviral load peaked at week 8 post infection (Fig. 3b). No significant differences in FIV DNA proviral loads were detected between vaccine groups over time.

#### Protocol II

At week 20 post vaccination, study animals were intravenously inoculated with FIV_PPR_ as detailed above. Quantitative PCR analysis of plasma detected FIV RNA in most study animals within 1 week of FIV inoculation (Fig. 3c). All CD134+SU vaccinated cats demonstrated FIV RNA in plasma by week 1 post FIV infection, and all sham and CD134 vaccinated cats were positive for FIV RNA by week 2 post infection. Although, the level of viremia (FIV RNA) decreased significantly over time in all groups (time *p* < 0.001, SI Table 1), no significant differences in FIV RNA levels were observed between vaccine groups. PBMC proviral DNA was detected in all study animals at week 1 post FIV infection (Fig. 3d) and was significantly elevated above baseline in all study groups by week 2 post inoculation. However, proviral loads did not differ significantly between groups over time in this phase of the study. Results indicate that vaccination with CD134 or CD134+SU complexes as prepared in either trial does not provide protection from FIV infection in vivo.

**Table 1:** Anti-SU IgG fraction neutralizes in vitro FIV infection whereas whole serum enhances infection

Treatment	Factors removed from Serum	Immune factors retained in serum	Effect on FIV replication in vitro
Whole serum Sham group	NA	Complement and heat labile factors	NA
Whole serum CD134 group	NA	Complement and heat labile factors anti-CD134 IgG anti-293S IgG	Enhancement
Whole serum CD134+SU group	NA	Complement and heat labile factors anti-SU IgG anti-CD134 IgG anti-293S IgG	Enhancement
Heat-treated serum Sham group	Complement (inactivated) Heat labile factors (inactivated)	NA	NA
Heat-treated serum CD134 group	Complement (inactivated) Heat labile factors (inactivated)	Anti-CD134 IgG anti-293S IgG	NA
Heat-treated serum CD134+SU group	Complement (inactivated) Heat labile factors (inactivated)	Anti-SU IgG anti-CD134 IgG anti-293S IgG	Inhibition
Serum enriched for anti-SU	Complement (inactivated) Heat labile factors (inactivated) anti-CD134 IgG anti-293S IgG	Anti-SU IgG	Inhibition
Serum enriched for anti-CD134	Complement (inactivated) Heat labile factors (inactivated) anti-SU IgG anti-293S IgG	Anti-CD134 IgG	NA
Serum enriched for anti-293S	Complement (inactivated) Heat labile factors (inactivated) anti-SU IgG anti-CD134 IgG	Anti-293S IgG	NA

**Whole serum from CD134 and CD134+SU vaccinated cats significantly enhanced FIV replication in vitro**. Heat inactivation of whole serum from CD134+SU vaccinated cats resulted in in vitro inhibition, suggesting enhancing factors in whole serum are heat-labile. Anti-SU antibodies generated in CD134+SU vaccinated cats exhibit significant individual or combined inhibitory effects on FIV replication following heat-treatment to remove heat-labile proteins in serum, while serum enriched for anti-CD134 and anti-293S fractions neither inhibited or enhanced in vitro infection

### Purified anti-CD134 and anti-SU antibodies inhibit FIV replication in vitro

#### Protocol II

**Fig. 4:**
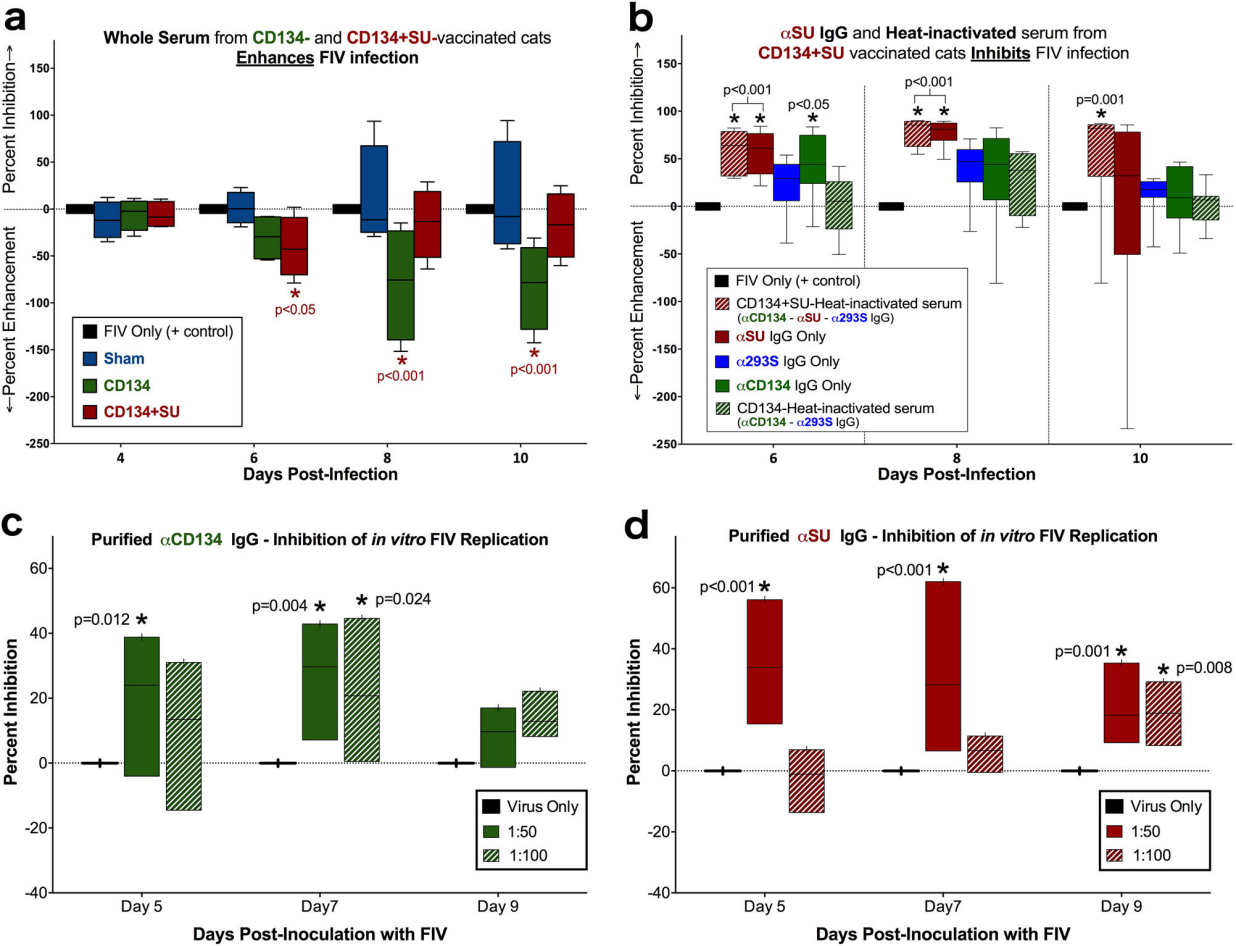
Anti-CD134 and anti-SU antibodies significantly inhibit FIV replication in vitro. **a** Whole serum from CD134 and CD134+SU vaccinated cats produced significant enhancement of FIV replication in GFox cells (CD134 *p* = 0.0005; CD134+SU *p* = 0.047), indicated by significantly decreased percent inhibition compared to FIV only infections. Sham vaccinated serum did not significantly inhibit infection. **b** Antibody fractions representing anti-CD134, anti-SU, and anti-293S were enriched or depleted from serum as described in the text, and analyzed for ability to inhibit in vitro viral infection. Significant inhibitory effects were observed with purified anti-SU IgG and heat-inactivated serum containing anti-SU, anti-CD134, and anti-293S IgG (Table 1). Enriched anti-CD134 (**c**) and anti-SU (**d**) antibodies recovered from serum of CD134 and CD134+SU vaccinated cats demonstrated significant inhibition of FIV replication in vitro (SI Table 2), confirming the individual capacity to inhibit FIV infection reported by Grant et al.^31^

Prior to FIV infection, serum was collected from sham, CD134 and CD134+SU vaccinated cats and incubated directly with FIV_PPR_ prior to in vitro inoculation. Additionally, serum from CD134+SU vaccinated cats was heat-treated (to inactivate complement) and depleted of anti-SU, anti-CD134, and/or anti-293S antibodies to evaluate the individual effects of these components on FIV replication in vitro. Results of these experiments are summarized in Table 1. Surprisingly, whole serum from both CD134 and CD134+SU vaccinated cats significantly enhanced FIV replication in vitro, as indicated by significantly higher FIV p24 ELISA absorbance values and decreased percent inhibition below threshold (Fig. 4a). In contrast, heat-inactivated serum depleted of anti-CD134 and anti-293S antibodies (containing only anti-SU antibodies) significantly inhibited FIV_PPR_ replication in GFox cell culture, as evidenced by significantly lower FIV p24 ELISA absorbance values and calculated percent inhibition compared to the FIV-only positive control (Fig. 4b). Similarly, serum containing only anti-CD134 antibodies inhibited FIV growth in vitro on day 6 of culture (Fig. 4b). Interestingly, a significant and sustainted inhibitory effect was observed at all timepoints utilizing heat-treated serum from CD134+SU vaccinated cats, in which complement was inactivated but contained all antibodies generated in vivo (anti-SU, anti-CD134, and anti-293S). In contrast, no inhibitory effect was observed in wells treated with heat-treated serum from CD134 vaccinated cats (complement-inactivated serum with anti-CD134 and anti-293S IgG). Serum depleted of anti-CD134 and anti-SU, but still containing anti-293S (i.e., contaminating) antibodies from CD134+SU vaccinated cats had a negligible effect of FIV replication, signaling that these irrelevant antibodies were not a cause of inhibition/neutralization interference in vitro. Importantly, enhancing effects were not observed for any of the heat-treated samples, regardless of antibody content, indicating that significant enhancement of FIV replication in vitro may occur as a result of heat-labile proteins such as complement.

Purified anti-CD134 and anti-SU antibodies from serum of vaccinated cats were used to further evaluate the individual capacity of these antibodies to inhibit FIV replication in GFox cells (SI Table 2). Analysis of mean absorbance values demonstrated that both purified anti-CD134 IgG (*p* < 0.01) and anti-SU IgG (*p* < 0.04) significantly inhibited FIV replication in vitro. Post hoc analysis revealed that both 1:50 and 1:100 dilutions of purified fractions of anti-CD134 (Fig. 4c) and anti-SU (Fig. 4d) were able to significantly inhibit FIV.

### Vaccinated cats exhibit divergent immunologic responses

**Fig. 5:**
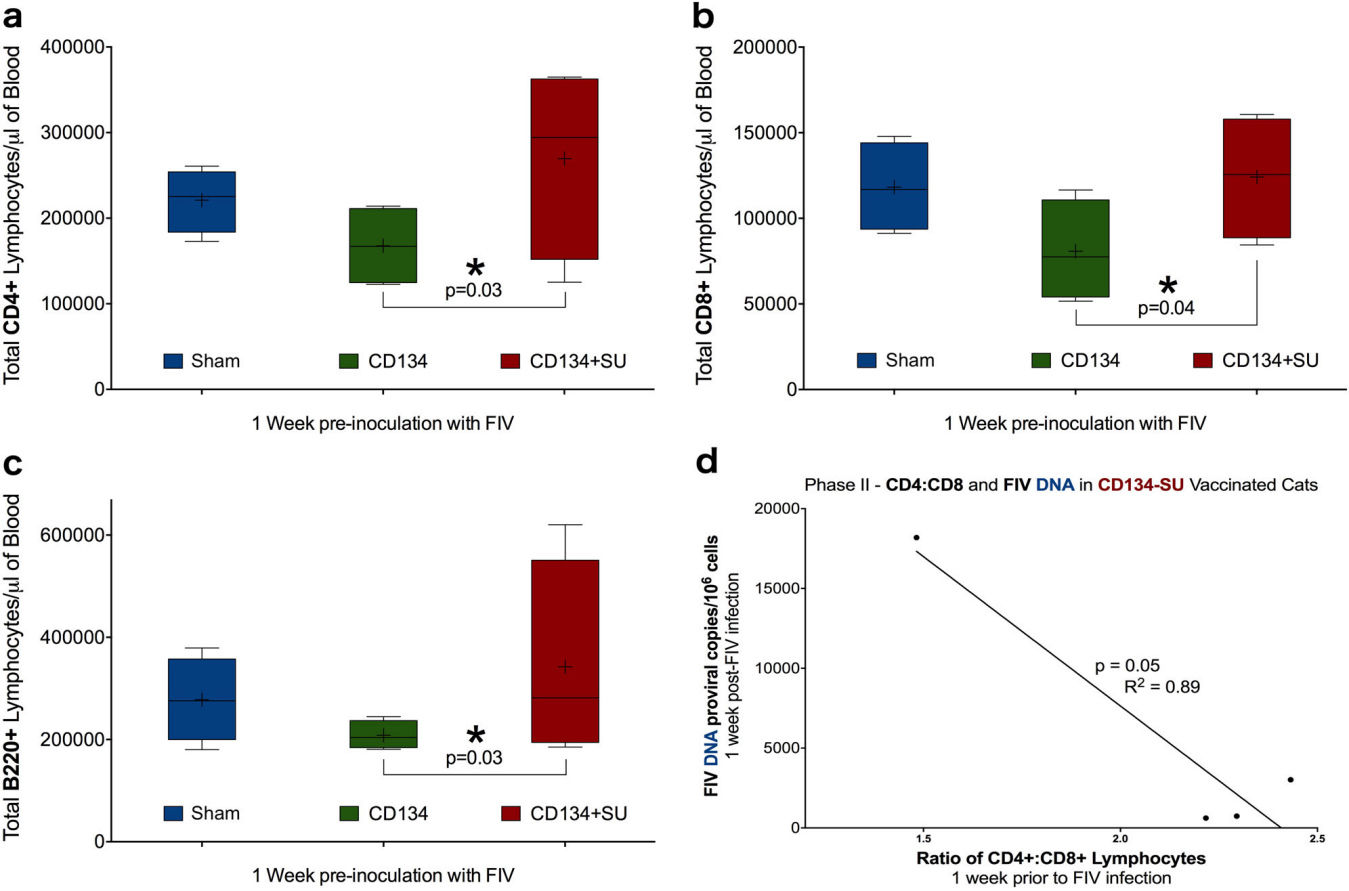
Immunization results in alterations in lymphoid immunophenotype in vaccinated cats. Prior to FIV challenge in Protocol II studies, CD8+ (**a**) CD4+ (**b**), and B220+ (**c**) lymphocytes were significantly elevated in CD134+SU vaccinated cats compared to CD134 vaccinated cats (*p* < 0.03, *p* < 0.04, *p* < 0.03, respectively). CD4+ and B220+ cell levels were slightly higher in CD134+SU vaccinated cats compared to Sham vaccinated cats prior to FIV challenge, and there was a trend for CD8+ cells to be decreased (*p* = 0.081) in CD134 vaccinated cats. **d** In CD134+SU vaccinated cats, decreased CD4:CD8 ratios prior to FIV challenge correlated with higher proviral DNA loads during acute stages of FIV infection, indicating that changes induced in circulating T-cell populations by vaccination-associated immunostimulation may predispose to enhanced susceptibility to viral infection (*p* = 0.05, *R*^2^ = 0.89)

In Protocol II, phenotype analysis of circulating immunocytes was performed to determine whether: (1) vaccine-associated changes in peripheral blood immunophenotype at the time of inoculation may have contributed to vaccine failure; or (2) vaccination protocols altered post-FIV challenge peripheral blood immune profile. Prior to FIV challenge, CD8+, CD4+, and B220+ lymphocytes were significantly elevated in CD134+SU vaccinated cats compared to CD134 vaccinated cats (*p* < 0.03, *p* < 0.04, *p* < 0.03, respectively) (Fig. 5a–c). Average numbers of CD4+ and B220+ lymphocytes also tended to be slightly higher in CD134+SU vaccinated cats than in sham vaccinated cats, but this finding was not statistically significant (Fig. 5a, c). Additionally, there was a trend (*p* = 0.081) for CD8+ lymphocytes to be decreased in CD134 vaccinated cats compared to sham vaccinated cats prior to FIV challenge (Fig. 5b). In CD134+SU vaccinated cats, the CD4:CD8 ratio prior to FIV challenge was correlated with early proviral load (*p* = 0.05, *R*^2^ = 0.89) (Fig. 5d), indicating that increased viral integration may be associated with decreased numbers of CD4+ lymphocytes relative to CD8+ cells as a result of vaccination.

## Discussion

The development of a partially effective commercial FIV vaccine, coupled with previous findings that anti-receptor autoantibodies are associated with better clinical outcomes, suggests that co-immunization with viral and receptor antigens might augment vaccine effectiveness. However, despite the promising results of in vivo production of neutralizing antibodies, cats in this study were not protected against homologous FIV infection, and occasionally exhibited transient enhanced infection (as evidenced by increased FIV RNA levels immediately post challenge). Several studies in FIV vaccine design have resulted in enhanced susceptibility to infection rather that protection.^26,28,30,37^ These complications are frequently paralleled in SIV and HIV vaccine development^37–43^ and have impeded progress in vaccine efficacy by a variety of mechanisms, including antibody-dependent viral enhancement or general immune activation.^25–30^ Accordingly, we performed several further analyses in this study in attempt to elucidate the mechanism for vaccine failure, including: (1) potential contribution of anti-SU or anti-CD134 vaccine-induced antibodies to enhanced FIV-replication; (2) assessment of alterations in circulating PBMC induced by vaccination (potentially in response to immune activation), which might render vaccinated cats more susceptible to viral challenge via expanded target cell population; (3) evaluation of antibodies generated in response to irrelevant antigens present in the vaccine preparation that could contribute to enhancement; and, (4) magnitude of relevant (i.e., anti-SU, anti-CD134) versus irrelevant (i.e., anti-huFc, anti-293S antigen) antibody responses. By determining which aspects of vaccination contribute to failure of this trial, future efforts can be modified to avoid pitfalls of this study.

Primary results of this study confirm that vaccination with soluble CD134-SU complexes induces in vivo production of both anti-SU and anti-CD134 antibodies, and when purified from serum of vaccinated cats, both antibodies are independently effective at neutralizing FIV infection in tissue culture. However, even in the presence of circulating neutralizing antibodies, cats challenged with homologous FIV virus were not protected against infection in vivo, and in some cases, succumbed to transient enhanced infection in the presence of SU rProtein. Correspondingly, in vitro analysis of whole serum from CD134+SU vaccinated cats indicated significantly enhanced viral infection in cell culture (Fig. 4a), but when heat-treated to inactivate complement and other heat-labile elements, serum containing anti-CD134 and anti-SU antibodies exhibited significant inhibitory effects. Indeed, heat-inactivated serum from CD134+SU (but not CD134) vaccinated cats significantly inhibited in vitro FIV replication at all-time points evaluated, indicating a sustained inhibitory effect when both anti-CD134 and anti-SU antibodies were present (Fig. 4b). These findings highlight the potential for a synergistic mechanism of viral inhibition between anti-CD134 and anti-SU antibodies produced in response to vaccination with CD134-SU complexes, but only in the absence of heat-labile serum factors.

Complement enhancement of viral infection has been previously reported in HIV^44–47^ and SIV^48–50^ studies, and may occur via both antibody-dependent and antibody-independent mechanisms of complement activation.^51–58^ Similar consequences of complement activation may be responsible for the enhancement effects noted in this study. For example, immunization with anti-receptor complexes may induce complement activation in vivo, resulting in viral opsonization or deposition of antibody-complement complexes on the cell to provide prolonged receptor contact and subsequent fusion within the cell membrane.^37,45,59^ Interestingly, non-heat-treated whole serum from sham vaccinated cats did not enhance FIV-replication in vitro (Fig. 4a), despite the presence of serum complement in treated wells. This suggests that in vivo complement activation may not occur in the absence of soluble CD134 or recombinant SU protein, and that the presence of anti-receptor complexes in vaccinated cats may in fact influence the activation of complement and enhancement of FIV replication in vitro. Other heat-labile factors, such as cytokines, chemokines, growth factors, and adhesion molecules, are also present in serum and may similarly contribute to enhanced replication of FIV in vitro,^60^ particularly if the vaccination protocol has altered the immune landscape. Additionally, because sham vaccinated whole serum did not cause enhancement effects like CD134 and CD134+SU vaccinated whole serum, it is reasonable to assume that activation of such viral enhancing elements in this study are likely attributable to vaccination with soluble CD134, recombinant SU protein, and/or purification by-products (huFc or 293S cells). Future studies will be directed at elucidating the role that complement activation and other heat-labile serum components play in the interference of the anti-receptor antibody response during viral infection.

An additional factor to consider is the disproportionate antibody response generated in vaccinated cats against the vaccine by-products; huFc and 293S cell contaminants co-purified with the recombinant proteins. Microsphere immunoassay results demonstrated a substantial quantity of anti-Fc antibodies in Protocol I vaccinated cats, as well as a marked anti-293S antibody response in Protocol II animals. While in vitro experimental results indicated that these antibodies did not directly interfere with or enhance FIV infection, it is possible that their production may have indirectly interfered with vaccine efficacy by compromising the neutralizing antibody response. Based upon the magnitude of antibodies generated to these antigens (huFc and 293S), the proportion of coincident anti-CD134 and anti-SU antibodies produced in response to the targeted immunogens was on average 10-fold lower, indicating that the bulk of antibody production was directed at these “irrelevant” antigens instead of their intended target. As such, the potential level of anti-CD134 and anti-SU antibody necessary for protection from FIV infection was likely never attained in vivo, thus preventing the current immunization strategy from providing a neutralizing antibody response strong enough to prevent infection in vaccinated cats. Moreover, the potential for these foreign antigens to activate complement and other heat-labile immune response factors may have contributed to the transient enhancement effects observed in vivo, and the magnitude of the antibody response to such antigens may account for the in vitro enhancement effects observed in cats vaccinated with anti-receptor complexes. Finally, it is possible that exposure to foreign/irrelevant antigens may have driven antibody production toward a predominant IgG1 response rather than a more protective IgG2a response. The potential for this may be subsequently avoided through use of non-alum based adjuvants such as Ribi, which enhance IgG2a production and the Th1 response, as well as production of protective interferon-gamma.^61–63^ Future vaccine design methods will incorporate the use of more highly purified immunoadhesion peptides which lack purification by-products, thereby limiting the potential for interference in neutralizing antibody production and innate immune activation by irrelevant antigens.

In SIV and HIV immunization studies, much of the capacity to enhance susceptibility to infection has been attributed to an increase in general immune activation and/or expansion of lymphoid target cells, and this feature has also been observed in FIV studies.^29,43,64–68^ Flow cytometry analysis of lymphoid immunophenotypes in Protocol II identified significant variations in CD4+, CD8+, and B220+ lymphocytes among CD134 and CD134+SU treatment groups prior to infection (Fig. 5). A positive correlation was observed between the CD4:CD8 ratio and acute DNA proviral load in CD134+SU immunized cats. While this result was statistically significant, it is based upon a small number of animals and the relevance of this finding would be strengthened by additional datapoints. Nevertheless, these results collectively indicate that an acute, vaccine-induced increase in CD4+ lymphocytes relative to CD8+ cells in CD134+SU immunized cats may contribute to viral integration and the establishment of FIV infection. However, no significant differences in immunophenotype prior to infection were observed between sham vaccinated cats and either CD134 or CD134+SU vaccinated cats in Protocol II of this study, suggesting that alterations in circulating immunophenotype due to vaccination with anti-receptor complexes may not play a major role in vaccine failure.

Alternatively, it is possible that vaccination with alum (aluminum hydroxide), a widely used immunopotentiating reagent frequently used in vaccine preparations to boost humoral immunity,^69–71^ resulted in increased amounts of target cells and general immune activation in this study, thus priming the immune system for infection prior to FIV inoculation. However, sham vaccinated cats also received alum during this study, and no evidence of viral enhancement was observed in these animals in vivo (Fig. 3) or through use of their serum in vitro (Fig. 4). These results suggest that any enhancing effect observed in SU-huFc vaccinated cats of Protocol I and CD134+SU vaccinated cats of Protocol II was less dependent upon the use of alum, and implicate the immunogen among these groups: SU rProtein. The direct indictment of SU as a sole cause of vaccine failure is, however, unlikely considering the following evidence: (1) all CD134 vaccinated cats in Protocol II became infected by week 1 post infection, yet these animals were not immunized with SU, and did not generate an anti-SU antibody response prior to infection; and (2) purified anti-SU antibodies from CD134+SU vaccinated cats significantly inhibited FIV replication in vitro, substantiating that the presence of these antibodies elicit protective effects against FIV infection as previously reported.^31^ Future studies could evaluate impacts of vaccine adjuvantation and by-products on PBMC susceptibility to FIV infection, and thus pave the way to assess mechanistic impacts of vaccine components on enhancement.

Vaccine failure occurred despite administration of a partially characterized vaccine inoculum that resulted in minimal to moderate infection. Development of stocks with well-characterized in vivo titrated viral infectivity would be required for additional studies determining low dose challenge efficacy, and we cannot completely rule out that vaccinated cats may have been protected against FIV infection with different viral inoculum.

Measurement of neutralizing antibodies via in vitro analysis that correlate with protection against lentiviral exposure is not straightforward.^72^ Indeed, while previous studies have demonstrated protection from infection with a dual-subtype, commercially available FIV vaccine, neutralizing antibody titers were not correlated with protection against viral challenge.^20,73^ In this study, we utilized a tissue culture indicator cell line to assess the capacity of vaccine-induced, FIV-specific antibodies to inhibit or enhance FIV replication in vitro as a means to exclude potential sources of vaccine-induced viral enhancement. The use of the GFox (CRFK) cell line did not accurately predict in vivo protection against challenge, and alternatively, the use of primary PBMC may have helped to better define actual in vivo protection afforded by vaccination.

In summary, the results of this study identify specific obstacles to overcome in the development of anti-receptor antibody immunization, and highlight potential targets for optimization and in vivo study design. Immunization with soluble receptors (CD134) and viral surface glycoprotein (FIV-SU) elicits in vivo production of neutralizing antibodies (anti-CD134 and anti-SU IgG) in vaccinated cats, both of which significantly inhibit FIV replication in vitro. Immunization with anti-receptor complexes did not prevent FIV infection, though efficacy may have been negatively affected by a substantially disproportionate antibody response to vaccine by-products that likely compromised an effective neutralizing antibody response. Importantly, the role of heat-labile factors, including complement, in serum of vaccinated cats may play a role in the interference of anti-receptor antibody function and vaccine efficacy, and vaccination protocols need to be developed that do not enhance populations of circulating cells with enhanced susceptibility to FIV infection. Collectively, these findings illustrate several key features of the receptor-mediated immunogenic response in cats, and suggests that alteration of immune landscape during vaccination may improve efficacy in the development of a lentiviral vaccine.

## Materials and methods

### Vaccine design

Two vaccine trials were conducted to test whether addition of Fe-CD134 antigen to the vaccine preparation would augment the efficacy of a vaccine that contained only FIV-Env. Protocol I vaccine trial included immunization with a FIV-PPR-SU-huFc immunoadhesin, either alone or together with soluble CD134-huFc as a complex. These proteins were expressed in and purified from Chinese hamster ovary (CHO) cells as previously described,^74^ and the human Fc tag, in-frame with either protein, served as a means to purify the proteins using Staphylococcus Protein A-Sepharose.^13,75^ Protocol II vaccine trial employed soluble, His-tagged CD134 and FIV-PPR-SU derived by expression and purification from human 293ST cells. Soluble feline CD134 (aa 28–215) with a C-term 6× histidine tag, and FIV-PPR-SU (aa 170–601) were cloned into pCMV mammalian expression vectors (Life Technologies, Carlsbad, CA) and transfected into human 293ST cells using Lipofectamine 2000 (Life Technologies). His-CD134 (CD134-293S) was purified by nickel chromatography and untagged PPR- SU (PPR-SU-293S) was purified using Galanthus lectin-sepharose (Vector Laboratories, Burlingame, CA) from supernatants of 293S cells grown in defined serum-free medium (FreeStyle medium, Invitrogen).

### In vivo protocols

#### Animal welfare

This study was approved by the Colorado State University Institutional Animal Care and Use Committee; 14-4872A—Molecular Characterization of FIV. Colorado State University’s animal care program is licensed by the United States Department of Agriculture (USDA), accredited by Association for Assessment and Accreditation of Laboratory Animal Care (AAALAC) International, and holds an Office of Laboratory Animal Welfare (OLAW) assurance (A3572-01). All animal experiments complied with the National Institutes of Health guide for the care and use of Laboratory animals (NIH Publications No. 8023, revised 1978). Prior to experimental procedures, all study animals were anesthetized by intramuscular injection of ketamine (20 mg/kg) and acepromazine (2 mg/kg) to minimize animal suffering and distress. All study animals were monitored daily by animal care personnel for development of clinical signs of FIV infection and observed by clinical veterinarians. No animals exhibited clinical signs associated with FIV infection or any other untoward conditions during vaccine and challenge experiments.

#### Protocol I

Fifteen (15), 8–11-week-old specific pathogen free (SPF) cats (Andrea D. Lauerman Specific Pathogen Free Feline Research Colony, Fort Collins, CO) were housed within barrier rooms in accordance with Colorado State University (CSU) IACUC-approved protocols at a CSU AAALAC-international accredited animal facility, and were acclimated to the facility for 2 weeks prior to the initiation of the study. An outline of the study design for Protocol I is presented in Suplementary Information (SI Fig. 1A). At weeks 0, 4, and 8, all cats were subcutaneously inoculated with 1 ml of vaccine composed of Dulbecco’s phosphate-buffered saline (D-PBS; Life Technologies Corporation, Grand Island, NY), 5 mg of Alum (aluminum hydroxide, an adjuvant licensed for use in cats that induces significant humoral vs cellular immune responses), and one of the following immunogens: (Group 1A) sham vaccine (D-PBS and Alum only)(sham; *n* = 5); (Group 1B) PPR-SU-huFc peptide-immunoadhesion (**SU**-huFc; *n* = 5); and (Group 1C) FIV-PPR-SU-huFc peptide-immunoadhesion complexed with soluble CD134 (**CD134+SU**-huFc; *n* = 5)). At week 12, all 15 cats were intravenously inoculated with 75,000 infectious units of FIV_PPR_ (1 ml of a viral stock solution with a TCID_50_ titer of 1 × 10^576^). Prior to viral challenge, this stock was tested in a pilot study to assess a dose that would provide a dose that would reliably result in low-level infection in the majority of unvaccinated cats. Five of eight cats inoculated mucosal and three of three cats inoculated subcutaneously with this dose developed low-level proviral loads (~500–2000 proviral copies per million PBMC, peak plasma viremia <500 particles/ml, data not shown). We therefore used the same dose to challenge cats via subcutaneous route. Blood samples were obtained for all cats at 7-day intervals using previously established protocols, beginning at week 0 and ending at week 24.^76,77^

#### Protocol II

Twelve (12), 8–11-week-old SPF cats (Andrea D. Lauerman Specific Pathogen Free Feline Research Colony, Fort Collins, CO) were housed as previously described above in accordance with CSU IACUC-approved protocols. An outline of the study design for Protocol II is presented in SI Fig. 1B, and cats were randomly separated into three groups (*n* = 4 each) prior to the onset of this phase of the study: (Group 2A) Sham vaccinated; (Group 2B) CD134 vaccinated; (Group 2C) CD134+SU vaccinated. At weeks 0, 4, and 8, eight (8) cats were subcutaneously inoculated with 1 ml vaccine composed of Dulbecco’s phosphate-buffered saline (D-PBS; Life Technologies Corporation, Grand Island, NY), 5 mg of Alum, and 100 μg of soluble **CD134**-293S. The remaining 4 cats were inoculated with a sham vaccine composed of D-PBS and 5 mg Alum (Group 2A). At weeks 12 and 17, four cats that had previously received the vaccine containing soluble CD134-293S were instead inoculated with a vaccine composed of D-PBS, 5 mg of Alum, and 100 μg PPR-SU-293S complexed with CD134-293S (Group 2C, **CD134+SU** vaccinated). Group 2B (**CD134**-293S vaccinated) and Group 2A (**sham** vaccinated) were vaccinated as previously described at weeks 12 and 17 (Fig. 2b). Blood samples were collected from all cats using previously established protocols at 7-day intervals, beginning at week 0 and ending at week 24.^76^^,77^ At week 20, all 12 cats were intravenously inoculated with 75,000 infectious units of FIV_PPR_ (1 ml of a viral stock solution with a TCID_50_ titer of 1 × 10^5^^76^).

### Quantification of vaccine-specific IgG antibody production

Evaluation of vaccine-specific antibodies was performed using previously established microsphere immunoassay (MIA) protocols involving conjugation of carboxylated magnetic microspheres (MagPlex® Microspheres, Luminex, Austin, TX) with FIV glycoprotein (SU_PPR_) and soluble CD134 recombinant proteins (rProteins).^78,79^ Following conjugation protocols, a hemocytometer was used to determine microsphere concentrations, and protein coupling was confirmed via incubation of microspheres with primary antibodies and/or PE-conjugated detection antibodies.^78^ Successful coupling was defined by a median fluorescence intensity (MFI) of >2000. All samples from FIV-infected and negative control cats were diluted 1:50 in assay buffer and then incubated in duplicate with ~2500 conjugated beads per well. All samples were assayed in conjunction with FIV-A and naïve reference samples diluted 1:50 in assay buffer, as well as four diluent control wells per experiment. The MFI was calculated from ≥100 microspheres per analyte per well (Bio-Plex™ Manager 5.0) and then used for data analysis. All reagent concentrations, volumes, incubation times, acceptable standard recovery, and data analysis were as previously described.^78,79^

#### Protocol I

Plasma samples from all study animals collected at weeks 4, 6, 8, 10, 13, 15, 18, and 22 (SI Fig. 1A) were evaluated by MIA to detect anti-CD134 and anti-SU IgG antibodies. To detect antibodies specific to CD134 and/or FIV-PPR-SU, and to limit interference and detection of background antibodies specific to the huFc tag used to purify the Protocol I vaccine immunoadhesion peptides, alternate immunoadhesion molecules without huFc were purified from 293S cells (**CD134-293S** and **FIV-PPR-SU-293S**) and conjugated to microspheres for use in all MIA assays involving samples from Protocol I of this study (SI Fig. 2A). Microspheres conjugated to huFc rProtein and albumin were included with all samples and served as internal controls. Additionally, samples were analyzed in parallel using microspheres conjugated to huFc-purified FIV-PPR-SU **(FIV-PPR-SU-huFc)** to assess background levels of anti-huFc IgG generated in response to vaccination. Levels of anti-huFc were estimated by subtracting levels of anti-SU IgG (as detected by SU-293S-conjugated microspheres) from the anti-huFc/anti-SU background levels detected with SU-huFc-conjugated microspheres (Fig. 3). Immunodepletion studies were not conducted in Protocol I.

#### Protocol II

Vaccine-specific antibodies were detected in plasma samples of study animals at weeks 10, 14, and 19–23 (SI Fig. 1B) as described above. Similar to Protocol I, SU and CD134 rProtein produced in CHO cells from Protocol I (**huFc-purified**; **CD134-huFc** and **FIV-PPR-SU-huFc**) were conjugated to microspheres and used in all MIA assays involving samples from Protocol II of this study to detect antibodies specific to CD134 and/or FIV-PPR-SU, and to limit interference and detection of background antibodies specific to vaccine by-products associated with the 293S-purified SU and CD134 (SI Fig. 2B). All samples were analyzed in parallel using microspheres conjugated to 293S-purified CD134 and FIV-PPR-SU to assess background levels of anti-293S IgG generated in response to vaccination. The presence of anti-293S antibodies was confirmed by capture ELISA at an absorbance of 450 nm in 96-well flat bottom plates coated overnight 293S cell supernatant at a concentration of 10 μg/ml diluted in 100 µl of 0.1 M carbonate buffer (7.5 g/L sodium bicarbonate, 2.0 g/L sodium carbonate, pH ~9.5). Immunodepleted serum samples were diluted 1:25 in ELISA diluent and incubated for 2 h at room temperature. Each well was then washed five times (TEN buffer + 0.2% Tween 20) and then incubated with Cappel™ horseradish peroxidase (HRP)-conjugated goat anti-cat IgG (MP Biomedicals, Santa Ana, CA) diluted 1:5000 in ELISA diluent with 5% mouse sera for 1 h at room temperature. Each well was then washed five times and incubated for 10 min with 3, 3′, 5, 5′ tetramethyl benzidine (TMB) substrate and peroxidase (Biolegend, San Diego, CA) at room temperature before adding 2.5 N H_2_SO_4_. Photometric measurements of absorbance were then recorded for each plate at 450 nm as previously described.^80^

### Detection and quantification of FIV viral RNA and proviral DNA in blood

Blood samples collected during Protocol I and Protocol II were analyzed by real-time polymerase chain reaction (PCR) analysis to quantify FIV proviral DNA and FIV*gag* RNA at all timepoints described above and illustrated in SI Fig. 1. Plasma was isolated from EDTA-treated whole blood following centrifugation and frozen at −70 °C until processing. Viral RNA was extracted from 140 µl plasma using a QIAamp Viral RNA Mini Kit (Qiagen, Valencia, CA) according to manufacturer’s instructions. Viral RNA from each sample was converted to cDNA using Superscript II (Invitrogen) in individual reactions with random hexamers (Invitrogen) and then treated with RNase Out (Invitrogen) prior to real-time PCR quantification. Peripheral blood mononuclear cells (PBMC) from all cats were purified on a Histopaque (Sigma, St. Louis, MO) gradient, washed, pelleted, and then frozen at −80 °C. Proviral DNA was extracted from PBMCs using a DNeasy Blood and Tissue Kit (Qiagen, Valencia, CA) prior to real-time PCR quantification.

Real-time PCR reactions were performed on a CFX96™ Real-Time PCR Detection System (Bio-Rad, Hercules, CA) to detect and quantify FIV proviral DNA in PBMCs and FIV *gag* RNA in plasma using previously described FIV-A primers and probes,^81^ and an iTaq™ Universal Probes Supermix (Bio-Rad, Hercules, CA) containing an antibody-mediated hot-start iTaq DNA polymerase. Copy number of viral RNA in plasma was calculated as previously described,^77^^,82^ implementing a standard curve generated by diluting FIV-PPR virus stock in naive cat plasma and analyzed by reverse-transcriptase quantitative PCR as outlined above. To quantify proviral DNA in PBMCs, a real-time PCR standard curve was generated from serial dilutions of feline PBMCs from 1000 to 5 × 10^6^ subjected to real time PCR for the cellular house-keeping gene, glyceraldehyde-3-phosphate dehydrogenase (GADPH) as previously described.^82,83^ Resulting proviral copy numbers were normalized to copies per 10^6^ cells based on the total amount of DNA present in the reaction (100 ng).

### Hematologic analyses

#### Protocol II

Complete blood counts (CBC) and serum biochemistry analysis were performed for all blood samples in Protocol II by the CSU Veterinary Diagnostic Lab (CSU-VDL). Blood was collected from all cats prior to the study to establish baseline values, then at each time point outlined above and in SI Fig. 1B. At weeks 20–24, the percentage of cells positive for CD4, CD8, Fas, and B220 surface antigens was determined by incubating 30 µl of EDTA-treated blood from each cat in 96-well round-bottom plates with 0.6 µl of RPE-labeled anti-feline CD4 (Southern Biotech; clone 3–4F4), FITC-labeled anti-feline CD8 (Southern Biotech; clone fCD8), PE/Cy7-labeled anti-feline CD45R/B220 (Biolegend; clone RA3-6B2), and APC/Cy7-labeled anti-feline Fas/TNFRSF6 (R&D Systems; clone 431006) mouse monoclonal antibodies diluted in FACS buffer (5% BSA, 0.1% sodium azide in PBS). Following incubation for 30 min in the dark at room temperature, red blood cells (RBCs) were lysed, and stained cells were fixed using a Beckman Coulter Q-Prep work station with 600 µl of 0.1% Formic Acid, 270 µl of 0.06 M Na_2_CO_3_ anhydrous, 0.25 M NaCl, 0.25 M Na_2_SO_3_, and 90 µl 1% wt/vol paraformaldehyde in 1× PBS. Flow cytometry was performed on a Coulter Gallios (Beckman Coulter Inc, Brea, CA) and results were analyzed using FlowJo® software (FlowJo, Ashland, OR). Immunophenotype cell counts were calculated as previously described^77,82^ and compared with CBC data to evaluate changes in circulating immunophenotype over the course of vaccination and subsequent FIV infection. All CD4, CD8, and CD45R/B220 antibodies were directly labeled by the manufacturer. Anti-Fas antibody was unlabeled but subsequently conjugated to APC/Cy7 using a APC/Cy7® Labeling Kit (Abcam).

### In vitro antibody inhibition and enhancement of viral replication

#### Protocol II

Duplicate cell cultures consisting of GFox cells (CrFK cells overexpressing CD134)^84,85^ were established in 48-well plates at 40,000 cells/well and allowed to attach at 37 °C overnight. GFox cell cultures were grown at 37 °C and 5% CO_2_ in 250 µl of culture medium composed of Dulbecco’s modified Eagle’s medium (DMEM) with GlutaMAX-1, 10% fetal bovine serum (FBS), and 1× penicillin-streptomycin (10,000 U/l penicillin and 10,000 μg/l streptomycin), as well as 1 μg/ml of Fungizone® (Amphotericin B; Life Technologies).^86^ At day 0, 10 µl of FIV_PPR_ stock (containing 50,000 infectious units) was incubated for 1 h at 37 °C with 230 µl and 10 µl of whole serum from sham vaccinated, CD134 vaccinated, or CD134+SU vaccinated cats collected at week 19 (1 week pre-FIV challenge). Following incubation, infected media were then added to cell culture plates, bringing the total volume to 500 µl (1:50 serum/FIV). Duplicate negative control (1:50 serum only, no FIV) and positive control (FIV only, no serum) wells were included for each sample. At days 4, 6, 8, and 10 post inoculation, 200 µl of supernatant was removed from each well, frozen at −80 °C, and replaced with 200 µl of fresh culture media. At day 10, the supernatant collected from each well and each time point was assayed by a previously described capture ELISA protocol to detect FIV p26 antigen at an absorbance of 450 nm in 96-well flat bottom plates.^80^ Percent inhibition was calculated from mean absorbance values (Abs) using the previously described formula ((*X*–*Y*)/*X*) × 100, where *X* is fraction of cells infected in the absence of serum (virus only positive control) and *Y* is the fraction of cells infected in the presence of various serum treatments.^87^

To elucidate the discrete effects of anti-SU, anti-CD134, and anti-293S antibodies generated in vivo in response to immunization, serum from vaccinated cats collected at week 19 (1 week pre-FIV challenge) was immunodepleted of anti-CD134 and anti-SU antibodies via serial passages over Actigel ALD agarose bead resin (Sterogene, Carlsbad, CA) that had been coupled to either CD134-huFc or FIV-PPR-SU-huFc rProteins per manufacturer’s instructions. Anti-CD134 and anti-SU immunodepleted serum was then filtered through a microcentrifuge column by centrifugation at 8000 r.p.m. for 5 min. Vaccinated cat serum was immunodepleted of anti-293S antibodies by incubating 5 mg of acetone-powdered 293S cells with 500 µl of serum at 4 °C overnight with gentle agitation, followed by centrifugation at 8000 r.p.m. for 5 min and pipet recovery of the immunodepleted supernatant. Immunodepleted samples and a subset of whole serum were depleted of complement by heat inactivation (56 °C for 30 min). Immunodepletion of anti-CD134, anti-SU, and anti-293S antibodies was confirmed by capture ELISA (as previously outlined) at an absorbance of 450 nm in 96-well flat bottom plates coated overnight with either CD134-huFc, FIV-PPR-SU-huFc, or 293S cell supernatant at a concentration of 10 μg/ml diluted in 100 µl of 0.1 M carbonate buffer (7.5 g/l sodium bicarbonate, 2.0 g/l sodium carbonate, pH ~9.5). ELISA absorbance values indicated statistically significant depletion of all targeted antibodies (two-tailed *t*-tests; anti-CD134 *p* < 0.0001; anti-SU *p* = 0.005; anti-293S *p* = 0.0002).

Duplicate cell cultures consisting of GFox cells (CrFK cells overexpressing CD134)^84,85^ were established in 48-well plates as described above. At day 0, 10 µl of FIV_PPR_ stock (containing 50,000 infectious units) was added to 480 µl of fresh culture media along with 10 µl (1:50 dilution) of various combinations of immunodepleted serum, with the contents of each well as follows: (1) No FIV (Negative Control), (2) FIV only (positive control), (3) Heat-treated CD134 vaccinated serum (to inactivate complement) (4) Heat-treated CD134+SU vaccinated serum (5) anti-SU serum, (6) anti-293S serum, and (7) anti-CD134 serum. Following incubation for 1 h at 37 °C, infected media were pipetted onto duplicate GFox cell cultures and incubated at 37 °C for 12 h, at which point all culture media was removed from each well, discarded, and replaced with 500 µl of fresh culture media. GFox cells were visually inspected daily by inverted light microscopy for evidence of cell growth, attachment, syncytial cell formation, detachment, and cell death. At days 6, 8, and 10 post inoculation, 200 µl of supernatant was removed from each well, frozen at −80 °C, and replaced with 200 µl of fresh culture media. At day 10, the supernatant collected from each well and each time point was assayed by FIV p26 ELISA and the Abs value used to calculate percent inhibition as previously outlined.

To further identify the individualized effects of vaccine-produced antibodies in serum of immunized cats, previously absorbed anti-SU and anti-CD134 antibodies from each serum sample were recovered from agarose bead resins by elution with Pierce™ IgG Elution Buffer per manufacturer’s instructions. Elutions containing purified anti-CD134 or anti-SU IgG were pooled separately and then stored in 100 µl of D-PBS at 4 °C. Duplicate GFox cell cultures were established in 48-well plates as previously outlined, and at day 0, 10 µl of FIV_PPR_ stock (containing 50,000 infectious units) was combined with either 10 µl or 5 µl (1:50 or 1:100 dilution) of either purified anti-CD134 or anti-SU IgG, and added to sufficient culture media to bring the total volume to 500 µl. Duplicate negative control (no virus) and positive control (virus only) wells were included for each sample. Following incubation for 1 h at 37 °C, infected media were pipetted onto GFox cells and incubated at 37 °C for 12 h, at which point all culture media were removed from each well, discarded, and replaced with 500 µl of fresh culture media. At days 5, 7, and 9 post inoculation, 200 µl of supernatant was removed from each well, frozen at −80 °C, and replaced with 200 µl of fresh culture media. At day 10, the supernatant collected from each well and each time point was assayed by FIV p26 capture ELISA protocol and percent inhibition was calculated from mean absorbance values (Abs) as previously outlined.

### Statistical analyses

All analyses were conducted in the program R v3.0.2 (www.r-project.org) using the ‘stats’ package or using GraphPad Prism 6.0 software (La Jolla, CA). *P*-values <0.05 were considered significant. Repeated measures ANOVA was utilized to evaluate the difference in viral RNA and proviral DNA (copies/ml) among the three vaccine groups (Naive, CD134, CD134+SU) over time. Viral RNA, proviral DNA, and were log transformed to achieve normality prior to analysis. Repeated measures ANOVA was utilized to evaluate differences in anti-CD134, anti-PPR-SU, and anti-Capsid IgG antibodies in serum from the three vaccine groups (Naive, CD134, CD134+SU) over time, and was also used to evaluate differences in FIV replication (Absorbance) and Percent Inhibition among the vaccinated groups over time. Logically, inhibition was not analyzed for the negative and positive groups owing to all values being 1 or 0 respectively.

### Data availability

All relevant data files are available at. https://figshare.com/s/81df369c01e155a68514.

## Electronic supplementary material

SI Figure 1

SI Figure 2

SI Table 1

SI Table 2
