# Supplementary figures and images for: FIV vaccine with receptor epitopes results in neutralizing antibodies but does not confer resistance to challenge
Source: NPJ Vaccines. 2018 Apr 30;3:16. doi: 10.1038/s41541-018-0051-y (PMC5928050; doi:10.1038/s41541-018-0051-y)

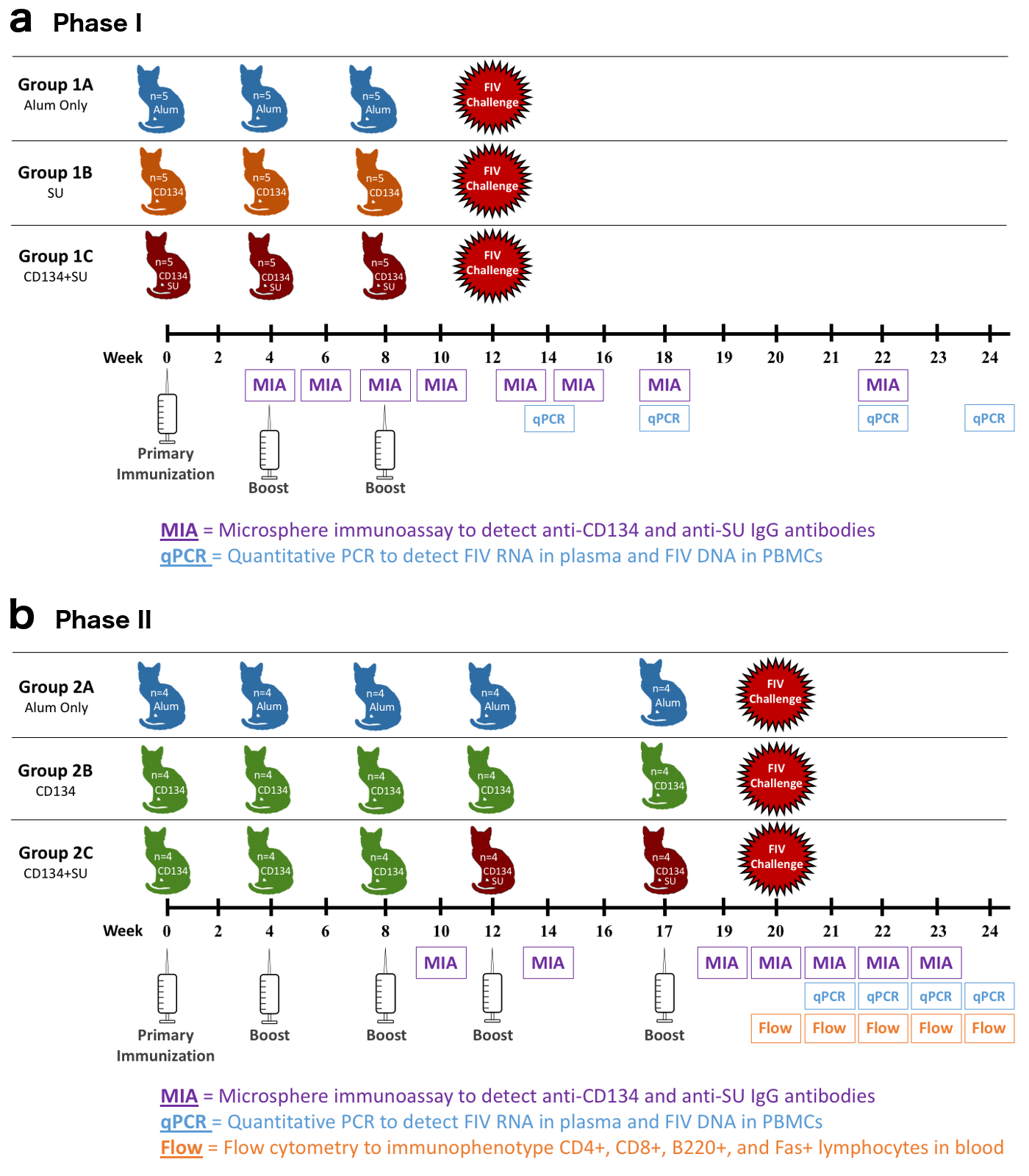

Supplement: Supplementary file 1 — SI Figure 1 [file 41541_2018_51_MOESM1_ESM.tif]

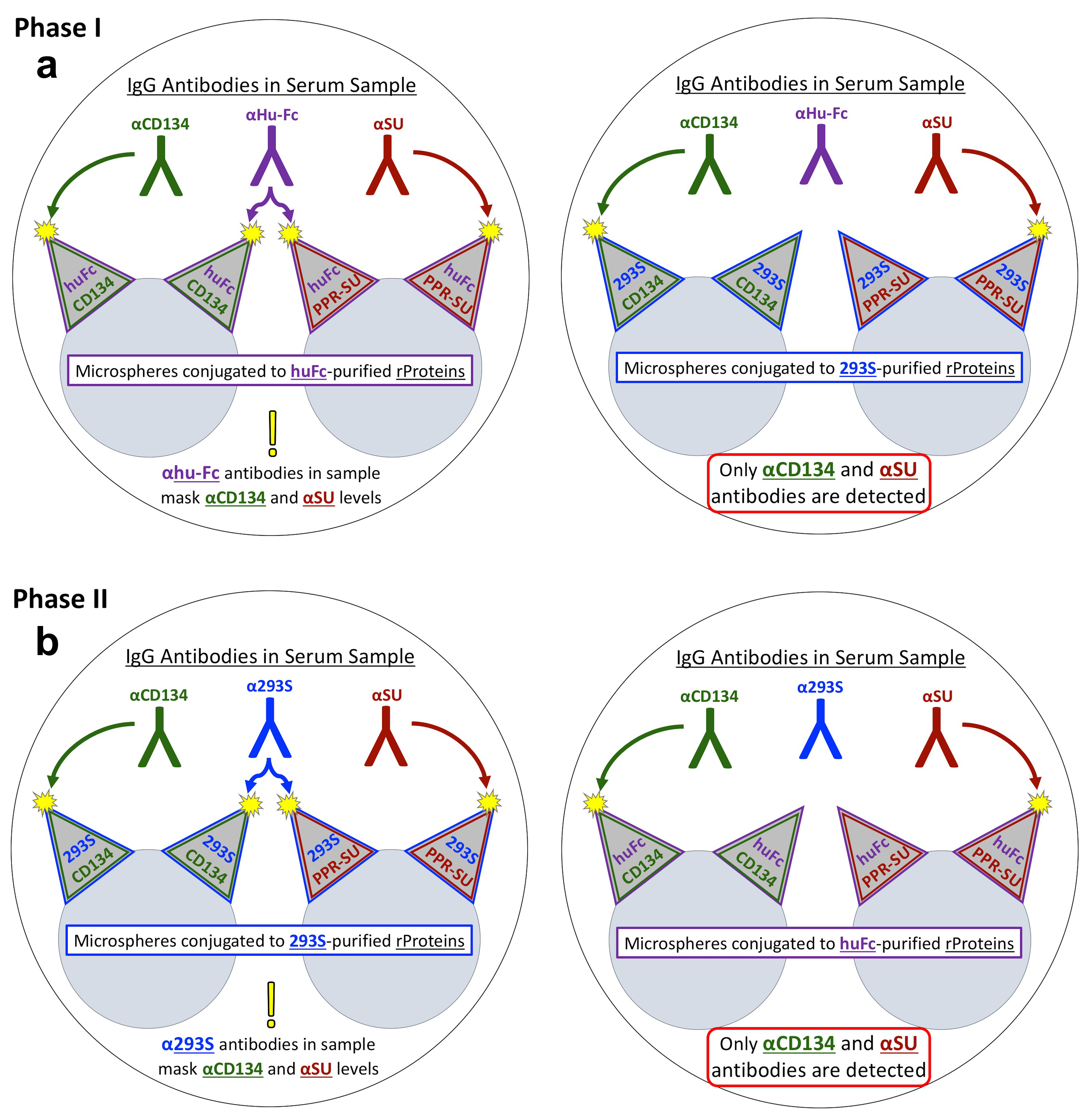

Supplement: Supplementary file 2 — SI Figure 2 [file 41541_2018_51_MOESM2_ESM.tif]
